# Notch signaling and progenitor/ductular reaction in steatohepatitis

**DOI:** 10.1371/journal.pone.0187384

**Published:** 2017-11-15

**Authors:** Carola M. Morell, Romina Fiorotto, Marica Meroni, Aileen Raizner, Barbara Torsello, Massimiliano Cadamuro, Gaia Spagnuolo, Eleanna Kaffe, Salvatore Sutti, Emanuele Albano, Mario Strazzabosco

**Affiliations:** 1 International Center for Digestive Health, Department of Medicine and Surgery, University of Milan-Bicocca, Monza, Italy; 2 Liver Center, Department of Internal Medicine, Section of Digestive Diseases, Yale University, New Haven, Connecticut, United States of America; 3 Department of Health Sciences, University “A. Avogadro” of East Piedmont, Novara, Italy; University of Navarra School of Medicine and Center for Applied Medical Research (CIMA), SPAIN

## Abstract

**Background and objective:**

Persistent hepatic progenitor cells (HPC) activation resulting in ductular reaction (DR) is responsible for pathologic liver repair in cholangiopathies. Also, HPC/DR expansion correlates with fibrosis in several chronic liver diseases, including steatohepatitis. Increasing evidence indicates Notch signaling as a key regulator of HPC/DR response in biliary and more in general liver injuries. Therefore, we aimed to investigate the role of Notch during HPC/DR activation in a mouse model of steatohepatitis.

**Methods:**

Steatohepatitis was generated using methionine-choline deficient (MCD) diet. For hepatocyte lineage tracing, R26R-YFP mice were infected with AAV8-TBG-Cre.

**Results:**

MCD diet promoted a strong HPC/DR response that progressively diffused in the lobule, and correlated with increased fibrosis and TGF-β1 expression. Notch signaling was unchanged in laser-capture microdissected HPC/DR, whereas Notch receptors were down regulated in hepatocytes. However, *in-vivo* lineage tracing experiments identified discrete hepatocytes showing Notch-1 activation and expressing (the Notch-dependent) Sox9. Stimulation of AML-12 hepatocyte-cell line with immobilized Jag1 induced Sox9 and down-regulated albumin and BSEP expression. TGF-β1 treatment in primary hepatic stellate cells (HSC) induced Jag1 expression. In MCD diet-fed mice, αSMA-positive HSC were localized around Sox9 expressing hepatocytes, suggesting that Notch activation in hepatocytes was promoted by TGF-β1 stimulated HSC. *In-vivo* Notch inhibition reduced HPC response and fibrosis progression.

**Conclusion:**

Our data suggest that Notch signaling is an important regulator of DR and that in steatohepatitis, hepatocytes exposed to Jag1-positive HSC, contribute to pathologic DR by undergoing Notch-mediated differentiation towards an HPC-like phenotype. Given the roles of Notch in fibrosis and liver cancer, these data suggest mesenchymal expression of Jag1 as an alternative therapeutic target.

## Introduction

The liver possesses a unique regenerative potential, which is differentially regulated according to the type of damage. In chronically damaged livers, when hepatocytes and cholangiocytes replication is impaired, functional repair relies upon the contribution of the hepatic progenitor cells (HPC) compartment. With persistent injury, HPC expansion occurs along with the recruitment of inflammatory mediators and the production of a fibrovascular stroma that sustains the newly formed tissue, giving rise to “ductular reaction” (DR), which is the hallmark of disease progression and fibrotic evolution[1–4].

HPC/DR expansion has been associated to liver fibrosis in several chronic liver diseases, including both Alcoholic and Non-Alcoholic Steatohepatitis (AH and NASH, respectively). Because of the increased prevalence of alcoholic liver disease and of risk factors for NAFLD/NASH (namely obesity, diabetes and metabolic syndrome) and their association with cirrhosis, liver failure and hepatocellular carcinoma (HCC), steatohepatitis is a growing health problem worldwide[5]. Furthermore, steatosis and steatohepatitis are commonly present in other conditions non associated with ALD or insulin resistance, such as iron deposition, hepatitis C, cystic fibrosis, etc. Because HPC/DR promotes disease progression and cirrhotic evolution, it is fundamental to unravel the mechanisms driving HPC activation in steatohepatitis.

Signaling pathways fundamental for liver morphogenesis are players in liver regenerative/reparative processes. Among them, Notch signaling is well suited to finely orchestrate the cross-talk among HPC and stromal cells; in fact Notch activation is mediated by direct homotypic or heterotypic cell-cell contacts. The ligands (Jagged [Jag]-1,-2, and Delta-like [Dll]-1, -3, -4) expressed on the “transmitting” cell activate Notch receptors (Notch-1 to -4) on the adjacent signal “receiving” cell; ligand/receptor interaction determines the cleavage of the Notch intracellular domain (NICD) by a γ-secretase. NICD associates with CBF1/RBPjK in the nucleus, allowing the transcription of several genes including the Hes and Hey-related family of transcription factors and SOX9. As a regulatory mechanism, Notch activation is carefully tuned by the specific ubiquitinase Numb, an endogenous inhibitor that targets NICD to the proteasome[6–8].

Notch is involved in liver development, physiology and pathophysiology[7, 8]. During development, Notch modulates the expression of liver-enriched transcription factors, with Sox9 recognized as the earliest Notch-regulated marker expressed by biliary committed hepatoblasts[9]. In liver regeneration, HPC express Notch-1 and -2 receptors which may bind to Jag1 positive neighboring mesenchymal cells[10, 11]. In mice lacking hepatic Notch-2 or RPB-jK and undergoing a biliary damage, HPC activation is severely impaired, suggesting that Notch is involved in HPC-driven liver repair and that Notch-2 is essential for tubulogenesis[11]. Furthermore, patients with Alagille Syndrome (AGS), a cholestatic cholangiopathy linked to Jag1 or Notch-2 deficiency, show an altered reparative response, with massive accumulation of cells with intermediate phenotype between cholangiocytes and hepatocytes[12]. These intermediate cells may derive from activated HPC unable to further differentiate or from hepatocytes undergoing a phenotypic switch; the histogenesis of HPC is not completely clear and there is evidence in favour of both hypotheses.

Altered hepatic Notch signaling is linked to liver carcinogenesis. Experimental models of constitutive activation of Notch receptors proved that Notch, in cooperation with other oncogenic pathways, leads to HCC and cholangiocarcinoma (CCA) development when aberrantly or ectopically expressed[13–17]. Accordingly, overexpression of Notch receptors has been described in human CCA[17], and a Notch signature was identified in a subset of HCC patients, along with hepatocytic Sox9 expression[16]. Interestingly, Notch activation in mature hepatocytes strongly correlates with a more malignant phenotype in hepatic cancer[15, 16, 18]. Notably, inhibition of Notch signaling with specific antibodies proved successful in decreasing tumor burden in a mouse model of liver cancer[19]. Furthermore it has been shown that intrahepatic CCA may derive from hepatocytes through Notch-mediated transdifferentiation[15].

In this study we aimed to understand the role of Notch in regulating HPC/DR response in a mouse model of steatohepatitis. We used a rodent model of steatohepatitis based on a methionine-choline deficient (MCD) diet that is not associated to insulin resistance and causes appreciable steatosis and inflammation[20], two conditions required for the development of HPC/DR. Our results show that HPC activation is linked to disease progression in terms of inflammation and fibrosis. Notch signaling appears to be activated in HPC/DR, in a subset of hepatocytes transitioning towards HPC-like cells (expressing Notch-1 intracellular domain and Sox9). Our data suggest that these cellular subpopulations establish a Notch-mediated cross-talk with mesenchymal cells that could be responsible for disease progression and eventually pave the way to liver cancer development in chronically damaged livers.

## Material and methods

### Animals and experimental protocols

8-weeks old C57BL/6 male mice were purchased from Harlan–Nossan. To induce NASH, mice were fed either methionine-choline deficient (MCD) (Laboratorio Dottori Piccioni) or control diet for 4 up to 8 weeks. Body weight was recorded weekly throughout the experiment. For hepatocyte lineage tracing experiments, R26R-YFP reporter mice were purchased from Jacksons Laboratories. To selectively induce reporter gene (YFP) expression only in mature hepatocytes, mice were infected with the hepatocyte-specific adeno-associated virus AAV8 that carries Cre recombinase under the control of the hepatocyte-specific promoter for thyroid-binding globulin (AAV8-TBG-Cre). Viral infection with AAV8-TBG-Cre (2.5X10*11 viral particles in sterile 1X PBS) was performed via retro-orbital injection as previously described[21]. One week after injection mice were fed with MCD diet as indicated. Mice were anaesthetized with sevofluorane and blood was collected by cardiac puncture. Livers were rapidly removed, tissues were harvested and some aliquots were snap frozen in liquid nitrogen whereas portions of each liver lobe were fixed in formalin and embedded in paraffin. All animal experiments were performed according to protocols approved by the Italian Ministry of Health, by the University Commission for Animal Care following the criteria of the Italian National Research Council, and by the Yale University Institutional Animal Care and Use Committee.

### In vivo Notch inhibition

To block Notch signaling during steatohepatitis progression, MCD diet-fed mice were treated with the γ-secretase inhibitor dibenzazepine (DBZ) via i.p. injection at a dose of 5μmol/Kg/day[11]. DBZ treatment was begun after 4 weeks of MCD diet, once steatohepatitis was established. MCD dietary regimen was kept throughout the treatment period with DBZ. Because of the toxicity of pan-Notch inhibition, mice were sacrificed 3 weeks after starting DBZ administration and tissues processed for histological analysis.

### Histology and immunohistochemistry

Tissue sections were stained by Hematoxylin and Eosin (H&E) to assess liver pathology. The severity of steatosis and lobular inflammation was scored by an experienced pathologist according to Kleiner[22]. Liver fibrosis was revealed by Sirius red histochemistry. Fibrosis extent was quantified with ImageJ software in 10 random micrographs (magnification 200x) by calculating the Sirius red positive area as the percentage of pixels above the threshold value with respect to the total pixels per area.

Paraffin embedded livers were analyzed for the following cellular markers: cytokeratin-19 (CK19, HPC/DR); Sox9 (biliary specific marker), αSMA (activated hepatic stellate cells [HSC] marker). Briefly, de-paraffinated sections were re-hydrated in alcohol and endogenous peroxidase activity was blocked with methanol/10% hydrogen peroxide. After unmasking with the proper antigen retrieval and treating with proper blocking solutions, the slides were incubated overnight at 4°C with the following primary antibodies against: CK19 (Troma III clone, DSHB; 1:100), Sox9 (Millipore; 1:100), αSMA (DAKO; 1:100), GFP (Abcam; 1:500, this antibody recognizes YFP). After rinsing, slides were incubated with the appropriate horseradish peroxidase-conjugated secondary antibody and developed with 3-3-diaminobenzidine (DAB). Alternatively, for immunofluorescence studies, slides were incubated with the proper fluorescent secondary antibody (Alexa Fluor 488, Life Technologies; 1:500; Alexa Fluor 594, Life Technologies; 1:500) and mounted with DAPI. The slides were analyzed with the Nikon Eclipse E800 microscope (Nikon) connected to a Nikon Sight DS-5Mc digital camera (Nikon).

CK19 quantification was performed in 5 random non-overlapping fields per slide (100x magnification) by calculating the CK19 positive (+ve) area with the software ImageJ (NIH) as percentage of pixels above the threshold value with respect to the total pixels per area. Similarly, to differentially quantify the distribution of CK19+ve cells in lobular versus portal areas, 10 random pictures (200X magnification) were taken and portal positivity was subtracted to total positivity to obtain lobular distribution.

For hepatocyte tracing experiments, the number of cells single positive for Sox9, CK19 and double positive for Sox9/YFP and CK19/YFP was evaluated in 20 fields for each control mouse (200X magnification) and in 10 fields for each treated mouse (200X magnification). A minimum of 490 or 830 of cells positive for the selected marker was examined for control and treated mice respectively. Results are represented as the percentage of Sox9 or CK19 positive cells co-expressing the YFP label.

### Laser capture microdissection (LCM)

LCM studies were performed to evaluate differential changes in gene expression specifically in CK19+ve cells (mainly HPC/DR) or in CK19 negative (-ve) parenchymal areas (mainly hepatocytes). Briefly, 10μm thick frozen liver sections were quickly stained with CK19 to specifically label HPC/DR, and microdissected under the MMI CellCut Plus laser capture microscope (MMI). After collecting CK19+ve cells/structures on a MMI isolation cap, CK19-ve areas from the same sample were selected in a different tube. Total RNA was extracted using RNeasy Plus Micro Kit (Qiagen) according to the manufacturer’s instructions, and reverse-transcribed with the High capacity cDNA reverse transcription Kit (Life Technologies). mRNA expression was quantified by real-time PCR (see supplementary section) following cDNA pre-amplification (TaqMan PreAmp Master mix, Life Technologies). Target genes-specific TaqMan probes were used for both amplifications.

### Nuclei isolation from paraffin embedded tissues

Fifty μm sections were cut and transferred into polypropylene tubes from each block to prepare a nuclear extract for staining and FACS analysis. Extraction of nuclei was performed using the method of Hedley et al.[23] with some modifications. Briefly, tissue sections were deparaffinized in xylene and rehydrated in an ethanol series. Tissue slices were incubated with an antigen retrieval citrate buffer (10 mM, pH6) for 2 hours at 80°C and with a pepsin solution (0.5%, pH1.5) at 37°C for another hour with intermittent vortex mixing. At the end of the incubation, cell debries were spun down at full speed centrifuge for 1 minute. Supernatans containing nuclei filtered through 40 μm nylon mesh, washed in PBS and aliquoted in conical bottom plates for staining and FACS analysis.

### Flow cytometric analysis

Nuclei isolated from paraffin embedded tissues were resuspended in PBS/5% BSA and incubated in ice for 30 minutes to block any nonspecific binding and were permeabilized using a cytofix/cytoperm kit (561651, BD Biosciences) for 20 min on ice. Then primary unconjugated antibodies against Sox9 (Millipore; 1:50), GFP (Abcam; 1:500, this antibody recognizes YFP) and N2ICD (c651.6DbHN clone, DSHB; 1:40) and conjugated Notch-1PE (mN1A clone, eBioscience;1:400) or their corresponding isotype controls in perm/wash were added to the corresponding wells. Following incubation for 30 min at 4°C in the dark, the cells were washed with perm/wash. Nuclei suspensions were then incubated with the appropriate fluorochrome-labeled secondary antibody (1:1000) and stained with DAPI (1:1000) in perm/wash buffer for 30 minutes at 4°C in the dark. After washing, pellets were resuspended in PBS in FACS tubes and were analyzed on a BD LSRII Flow Cytometer (BD, NJ). After the esclusion of debris and doublets, the DAPI positive population was analyzed for the expression of YFP, NICD1, NICD2 and Sox9. Expression of NICD1 or NICD2 in combination with Sox9 was evaluated in the YFP positive nuclei. Data were analyzed with FlowJo.

### *In vitro* treatments

We performed *in vitro* studies using the hepatocyte cell line AML-12 (ATCC). To evaluate the effects of Notch and Hedgehog (Hh) persistent activation in hepatocytes, AML-12 cells were stimulated with immobilized Jag1 (5μg/ml; R&D System), to reproduce cell-cell contact required by Notch signaling or recombinant Sonic hedgehog ligand (1000ng/ml; R&D System), to directly activate Hg pathway; Notch and Hh activation were carried out for 24 and 48 hours. In other experiments, primary cultures of mouse HSC and LX2 (a human HSC line) were treated with increasing doses of TGF-β1 (1, 5 and 10ng/mL) for 4 days or 72hrs, respectively.

### Gene expression analyses by quantitative real-time RT-PCR

RNA was extracted either from cell cultures or from total liver lysates with Trizol reagent (Applied Biosystems) according to the manufacturer’s instructions. 1 *μ*g of total RNA was retro-transcribed using the High Capacity cDNA Reverse Transcription Kit (Applied Biosystems). Realtime PCR was performed in a ABI 7900 thermocycler (Life Technologies), using TaqMan Gene Expression Master Mix and TaqMan probes for mouse Notch-related factors (Notch1 and -2, Jag1, Dll4, Hes1, Hey1, Numb), TNF*α*, CD11b, TGF-β1, Procollagen α1(I) (COL1(A1)), Sox9, HNF1β, Albumin and Bsep/Abcb11. Experiments on mesenchymal cells (expression of Jag1, COL1(A1), α-SMA) were performed with the SYBR green technique. Data were normalized to the Gapdh and/or Hprt gene expression. The results are expressed as 2^^-dCt^ or fold increase as indicated in bar graphs.

### Statistical analysis

Results are shown as means ± SD. Statistical comparisons were made using Student t-test or one-way analysis of variance (ANOVA) test with Tukey’s correction for multiple comparisons where appropriate. Correlation studies were performed using Pearson’s coefficient with two-tailed distribution. Statistical analysis was performed using Prism7; p values<0.05 were considered significant.

## Results

### MCD diet induces HPC/DR expansion throughout the parenchyma along with inflammation and fibrosis

**Fig 1:**
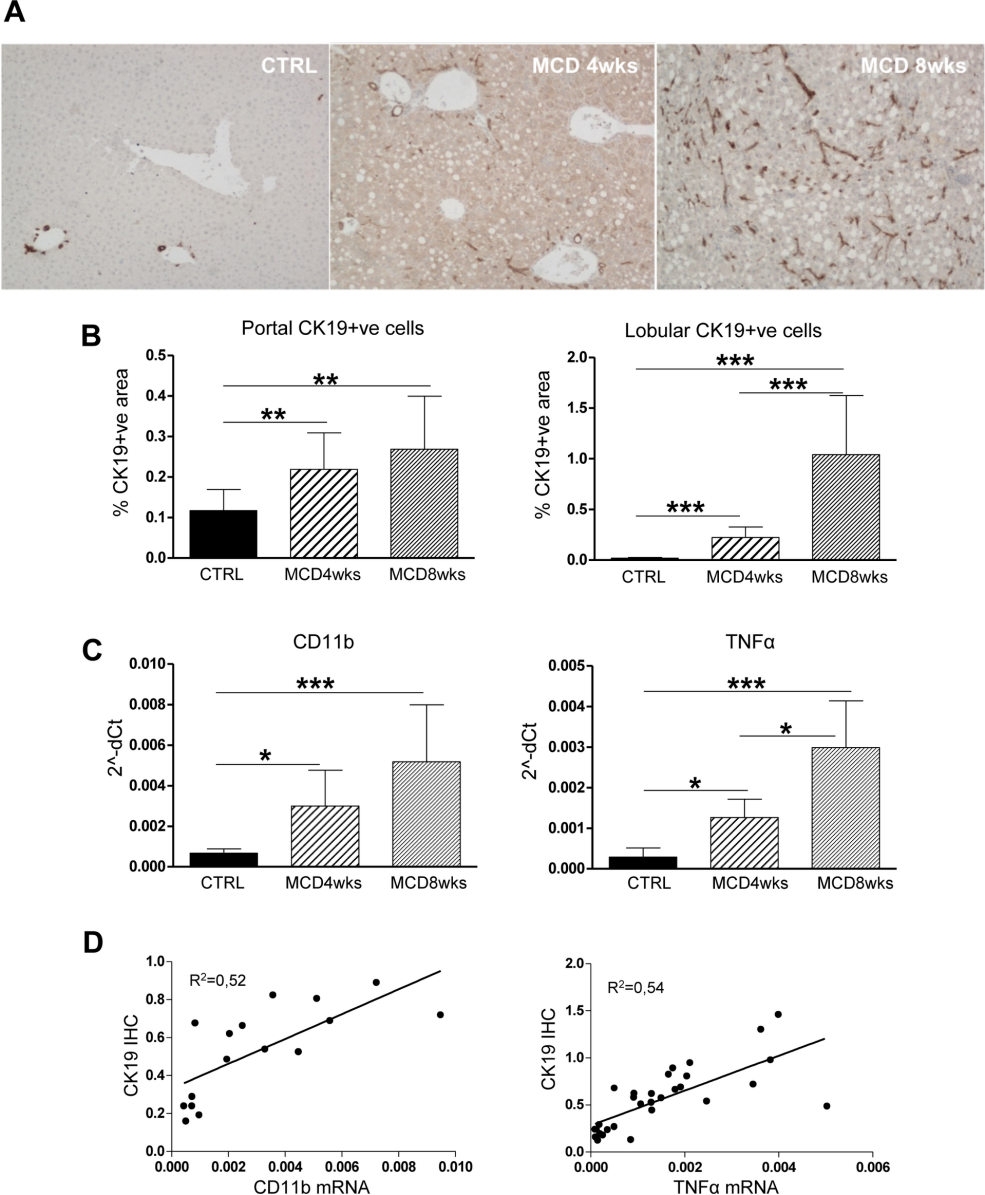
MCD diet induces HPC response and inflammation. **(A)** Immunohistochemical analysis on mouse liver specimens shows a progressive expansion into the liver parenchyma of the HPC compartment (CK19+ve cells) in mice treated with MCD diet respect to untreated controls. **(B)** The progressive spread of HPC from portal areas into the hepatic lobules was further confirmed by morphometric analysis. CK19 positivity in portal areas increased after 4 weeks of MCD diet and persisted throughout the treatment, whereas in the lobule the percentage of CK19+ve cells was significantly higher by the 8^th^ week of diet as compared to CTRL and MCD4wks groups. (n = 10–11 mice per group; *p<0.05, **p<0.01; ***p<0.001; CTRL = control, MCD4wks = MCD diet for 4 weeks; MCD8wks = MCD diet for 8 weeks). Original magnification: 100X. **(C)** Real time PCR analysis shows that MCD diet stimulated the inflammatory process, as confirmed by the mRNA expression of CD11b and TNFα, which positively correlated with the increase of CK19+ve cells **(D)** (n = 6–11 mice per group; *p<0.05, **p<0.01; ***p<0.001).

HPC/DR expansion in the liver of mice with steatohepatitis was assessed using cytokeratin-19 (CK19), a well-established marker. As shown in Fig 1, the extension of HPC/DR increased steadily in MCD diet-fed mice through the 8 weeks of treatment. Moreover, the pattern of CK19+ve cell distribution changed with the progression of hepatic damage, as, at the 4^th^ week of treatment, HPC/DR cells were mainly localized around portal areas, while, by the 8^th^ week they appeared to be spread into the liver lobule where they established intimate contacts with fat-laden hepatocytes (Fig 1A). Differential quantification of CK19 positivity in portal versus lobular areas showed that portal DR increased by 2-folds within 8 weeks of diet, whereas lobular CK19 positivity expanded by more than 10 times (Fig 1B).

In the context of liver diseases, DR is generally associated with inflammation. By investigating the expression profile of inflammatory markers we observed that gene expression of leukocyte activation markers CD11b and of TNFα was both induced by approximately 4 to 7-folds after MCD diet-feeding for respectively 4 and 8 weeks (Fig 1C) and correlated with CK19 positivity (Fig 1D).

**Fig 2:**
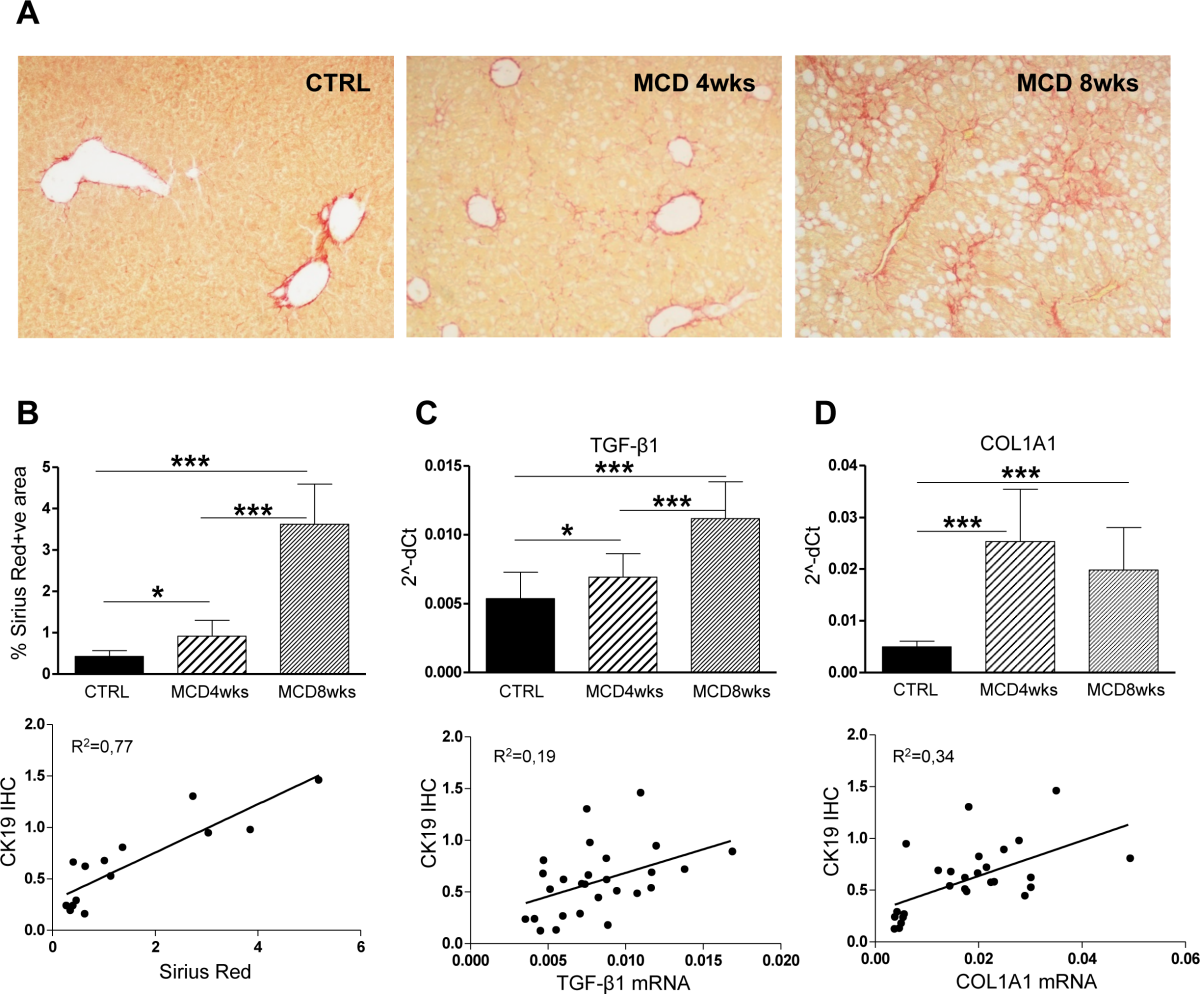
Fibrosis increases in MCD diet-induced steatohepatitis and correlates with HPC activation. **(A)** Histological analysis on Sirius Red stained livers revealed a progressive collagen deposition in MCD diet-treated mice from the 4^th^ to the 8^th^ week of treatment, as confirmed by morphometric quantification (**B**, upper panel). Collagen deposition strongly correlated with CK19 positivity (**B**, lower panel). MCD diet also stimulated the mRNA expression of profibrogenic mediators TGF-β1 and COL1A1(**C, D** upper panel, respectively), both of which correlated with increased HPC response (**C**, **D** lower panels) (n = 6–11 mice per group; *p<0.05, **p<0.01; ***p<0.001). Original magnification: 100X.

In parallel with the expansion of DR we observed a significant increase in hepatic fibrosis, as evaluated by collagen staining with Sirius Red (Fig 2A and 2B). Positive correlation was also evident between Sirius Red and the percentage of CK19+ve cells (Fig 2B). DR/HPC distribution resembled collagen deposition pattern, further indicating a functional link between CK19+ve cells and collagen-producing cells (cf. Figs 1A and 2A). Consistent with this observation, TGF-β1 and procollagen-1 (COL1A1) were significantly induced by MCD diet (Fig 2C and 2D, upper panels) and positively correlated with the presence of CK19+ve cells (Fig 2C and 2D, lower panels), again supporting the association between DR and liver fibrosis. The correlation of both the inflammatory and the fibrotic processes with CK19 positivity is consistent with a link between the enrichment in CK19+ve cells and the worsening of MCD diet-induced liver injury.

### Notch signaling in MCD diet-induced steatohepatitis

**Fig 3:**
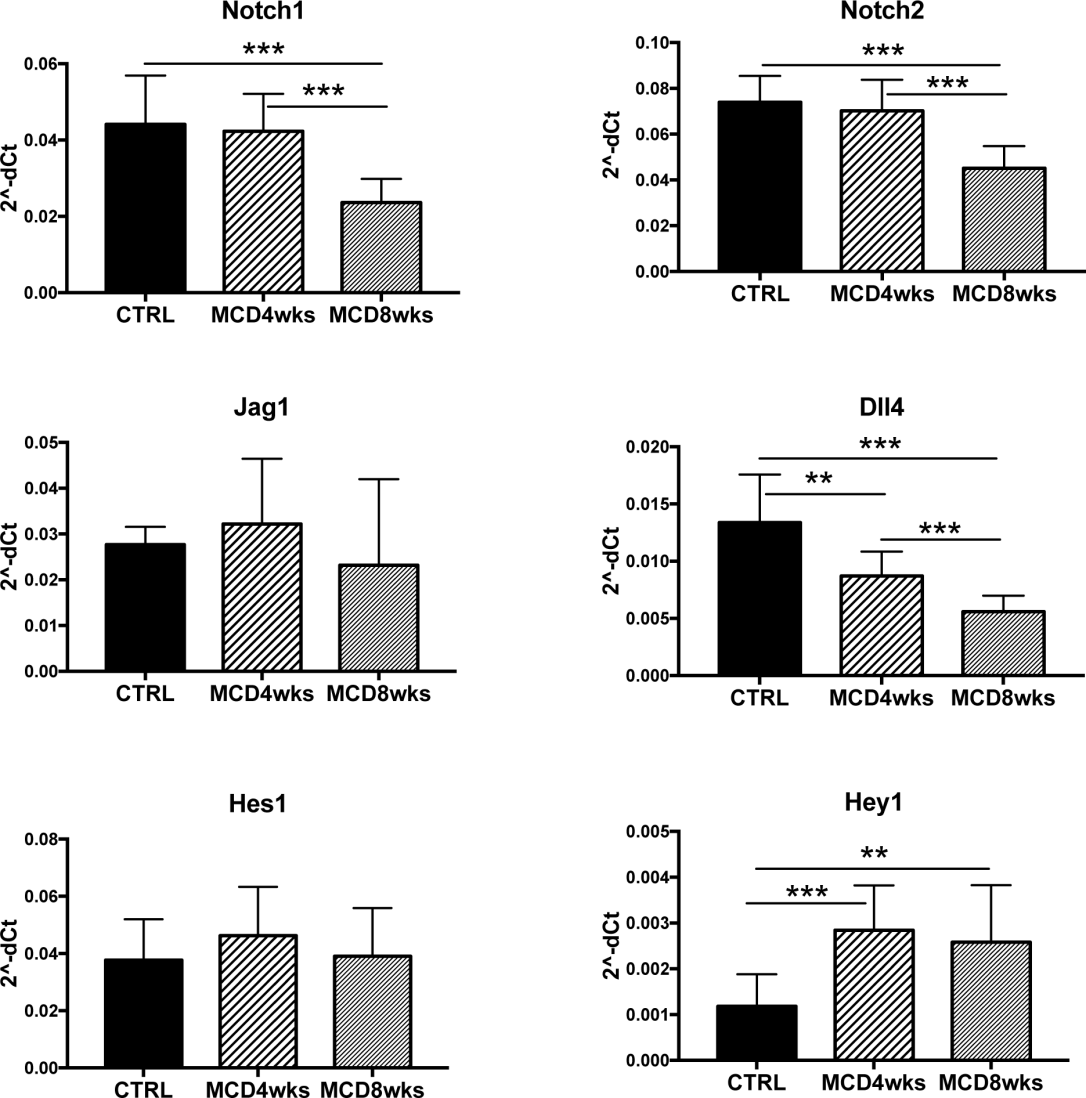
Gene expression analysis on total liver lysates of Notch signaling components. Real-time PCR analysis of Notch signaling components performed on total liver lysates revealed an overall downregulation of the expression of Notch receptors (Notch-1 and -2) and the ligand Dll4, without reducing the expression of the Notch ligand Jag1 or of the Notch-targets Hes1 and Hey1. Data are expressed as 2^-dCt^ (n = 10 mice per group; *p<0.05, **p<0.01; ***p<0.001).

Along with Hg and Wnt, Notch-1 and Notch-2 mediated signaling plays a primary role in regulating DR/HPC-driven repair in chronic biliary injuries[4, 10, 11]. To understand whether Notch signaling was involved in HPC/DR repair in the MCD model as well, we first performed gene expression analysis on total liver lysates (Fig 3). We found that the expression of Notch receptors Notch-1 and -2 (and of Dll4 ligand) significantly decreased with worsening of the disease, whereas the expression of the Jag1 ligand remained constant. The Notch target gene Hes1 did not change, whereas Hey1 showed a significant increase after 4 and 8 weeks of MCD diet.

**Fig 4:**
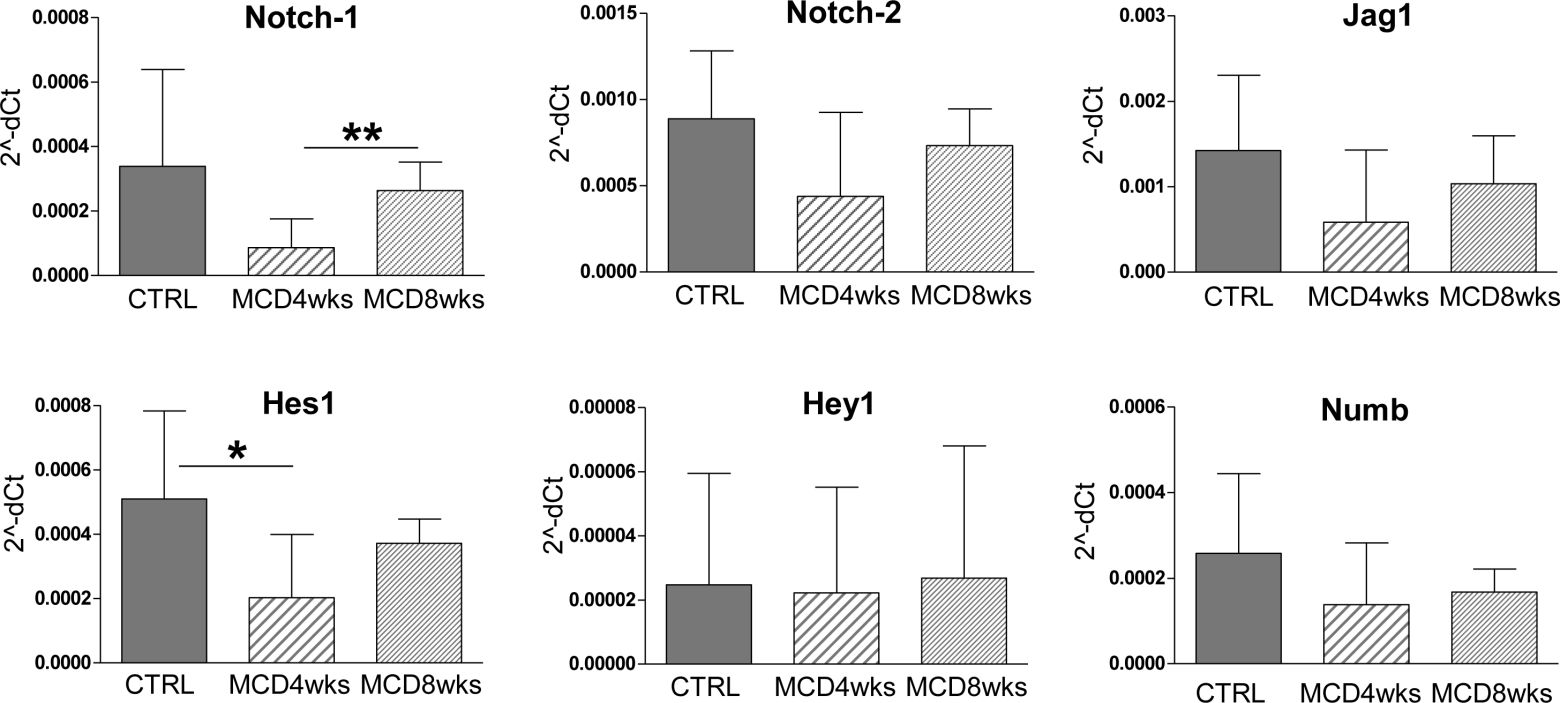
Notch signaling is not modulated in the CK19+ve population by MCD diet. The expression of Notch-related factors was evaluated on laser-capture CK19+ve microdissected samples, which comprise HPC/DR and biliary cells. Expression of the Notch pathway components was not significantly influenced by MCD diet in the CK19+ve population overall. Of note, Numb expression was not altered in these cells. Data are expressed as 2^-dCt^ (n = 5 mice per group; *p<0.05, **p<0.01; ***p<0.001).

**Fig 5:**
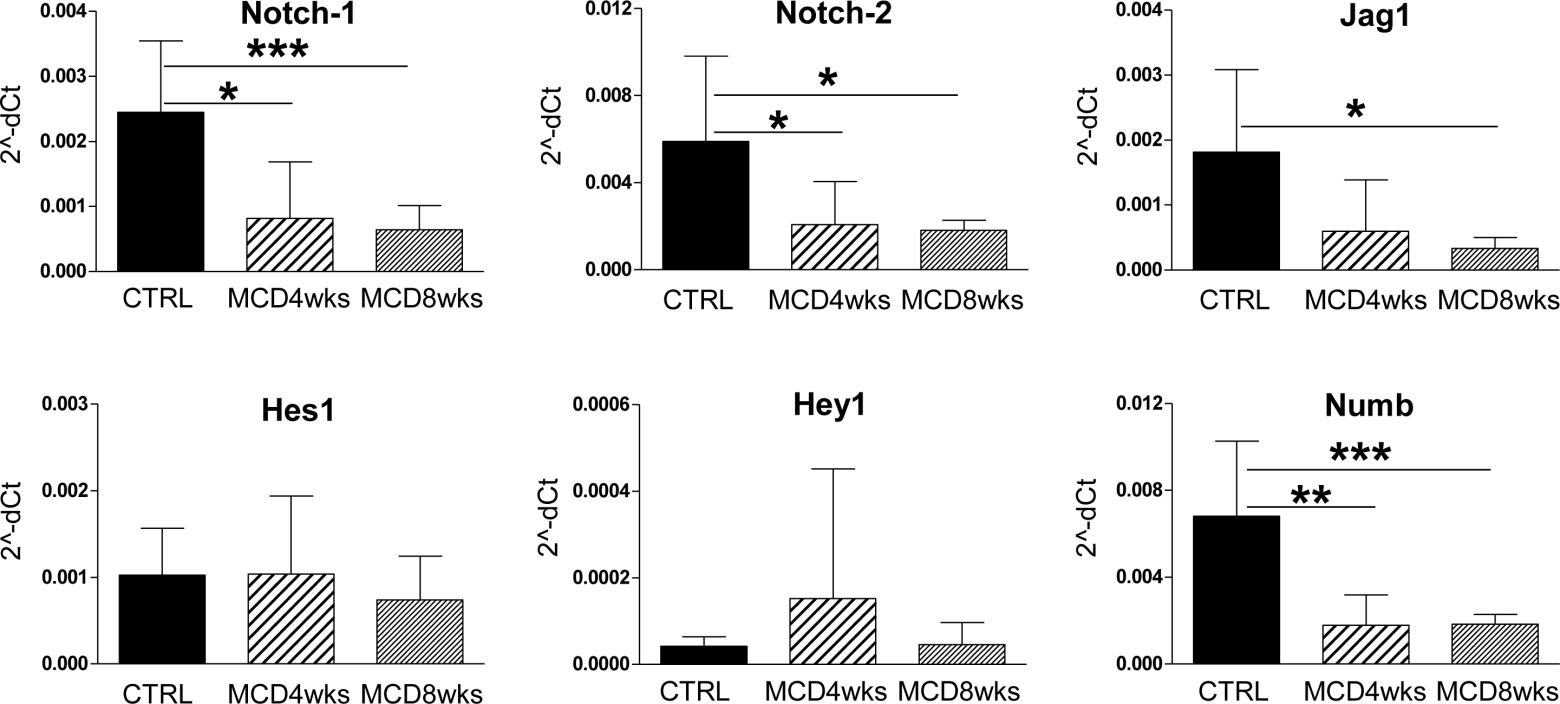
Notch signaling is modulated in laser-microdissected CK19-ve population. Real time PCR analysis of Notch components on CK19-ve microdissected samples showed a significant reduction, induced by MCD diet, of Notch receptors (-1 and -2) and Jag1 ligand expression in hepatocytes, respect to controls; on the contrary, the expression of Notch target genes was not altered. The expression of the Notch endogenous inhibitor Numb was strongly downregulated in hepatocytes by MCD diet. Data are expressed as 2^^-dCt^ (n = 5 mice per group; *p<0.05, **p<0.01; ***p<0.001).

Gene expression analysis on total liver samples likely reflects changes in the hepatocyte compartment since they represent the overwhelming cell population in whole liver lysates. Thus, to better define the cell type-specific expression of Notch factors, we used laser capture microdissection (LCM) to select CK19+ve areas and CK19-ve areas. In CK19+ve cells the expression of Notch related factors (i.e Notch-1, Notch-2, Jag1, Hes1, Hey1, Numb) did not significantly change during MCD diet treatment (Fig 4). On the other hand, the expression of Notch receptors and Jag1 ligand in the CK19-ve population (mainly hepatocytes) was significantly reduced (Fig 5). As recent reports indicate that Notch deletion improves glucose tolerance and reduces hepatic glucose production and lipogenesis[24, 25], we speculate that the down-regulation of Notch receptors in our mouse model could represent a counter-regulatory mechanism.

However, in the CK19-ve population (hepatocytes) we also noted a significant reduction of Numb (Fig 5), a negative regulator of Notch signaling that binds NICD and prevents its access to the nucleus [10, 26] suggesting that the decrease in Numb-mediated endogenous inhibition counterbalances the effects of decreased expression of Notch receptors. In fact, in spite of the strong down-regulation of receptors in these cells the expression of the target genes Hes1 and Hey1 remained substantially unchanged.

#### Notch signaling is activated in a subpopulation of hepatocytes

**Fig 6:**
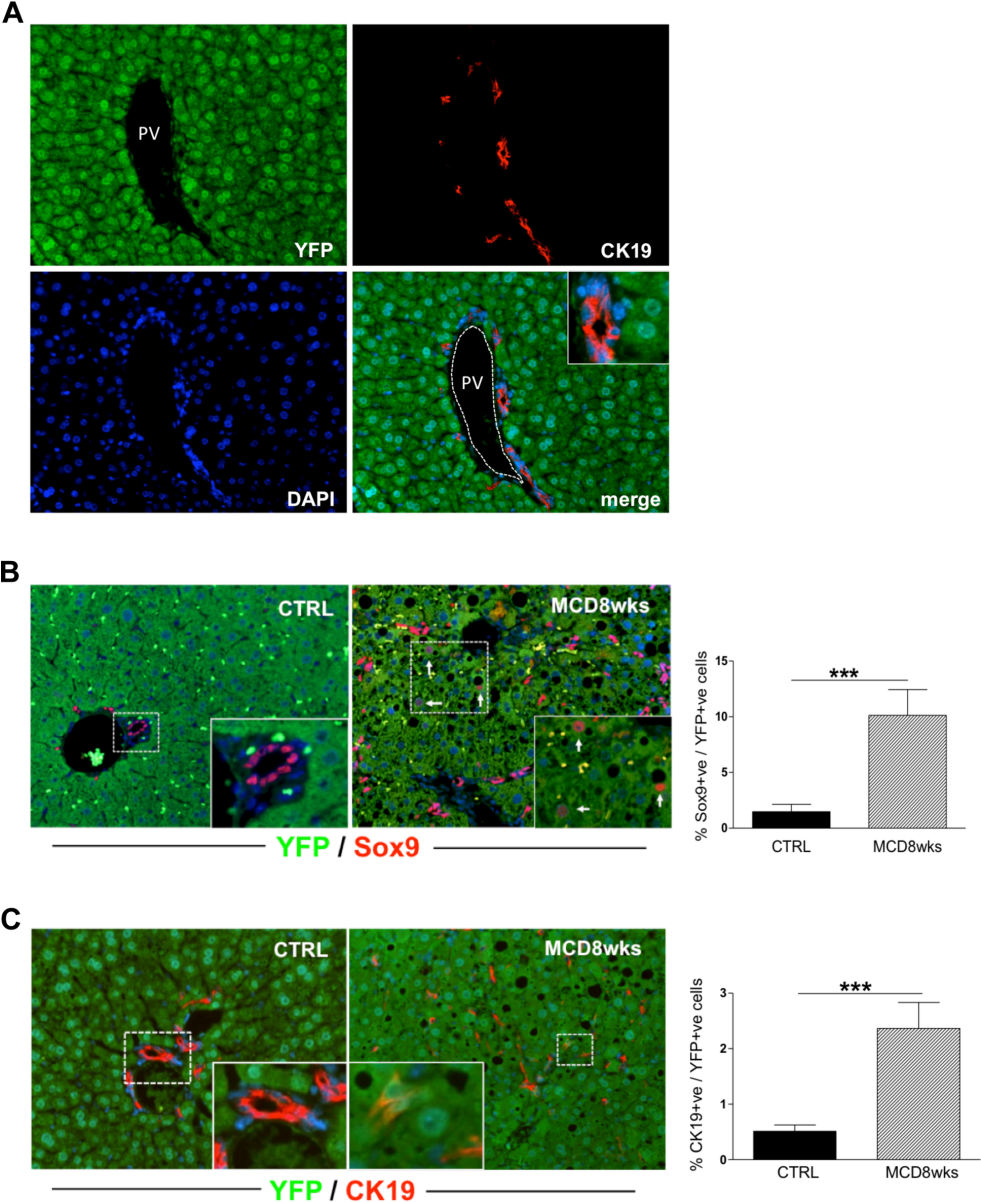
Lineage tracing experiments show hepatocytes conversion to HPC-like cells promoted by MCD diet-treatment. **(A)** R26R-YFP reporter mice were infected with AAV2/8 viruses containing Cre recombinase under the control of the hepatocyte-specific thyroid binding globulin (TBG) promoter (AAV8-TBG-Cre) to specifically and permanently label the hepatocyte cell lineage. The images depict YFP^+^ lineage-labelled cells 8 weeks after injection. Efficient and specific marking of hepatocytes with no marking of BECs (CK19^+^ cells) was observed. PV: portal vein. Fate tracing experiments were performed in R26R-YFP mice infected with AAV8-TBG-Cre. After MCD treatment, approximately 10% of hepatocytes (YFP, green) expressed Sox9 (red) **(B)**, and about 2% of YFP+ve/CK19+ve cells appeared in liver lobule **(C)**. The presence of Sox9 and CK19+ve hepatocytes was negligible in controls, as showed by immunofluorescent staining and histological analysis (n = 4–8 mice per group; *p<0.05, **p<0.01; ***p<0.001). Original magnification: 200X, insets 400X.

To better understand the pattern of Notch activation in hepatocytes, we next performed fate tracing experiments[21]. Hepatocytes were labelled *in vivo* by infecting R26R-YFP reporter mice with AAV8-TBG-Cre before inducing liver injury. The high affinity of AAV8 for hepatocytes[27] together with the expression of Cre recombinase under the liver specific TBG promoter allows a highly efficient and selective expression of Cre recombinase in hepatocytes only[21, 28]. Indeed, viral infection induced Cre-mediated YFP expression in more than 99% of hepatocytes, and YFP positivity was never detected in cholangiocytes lining bile ducts (Fig 6A).

**Fig 7:**
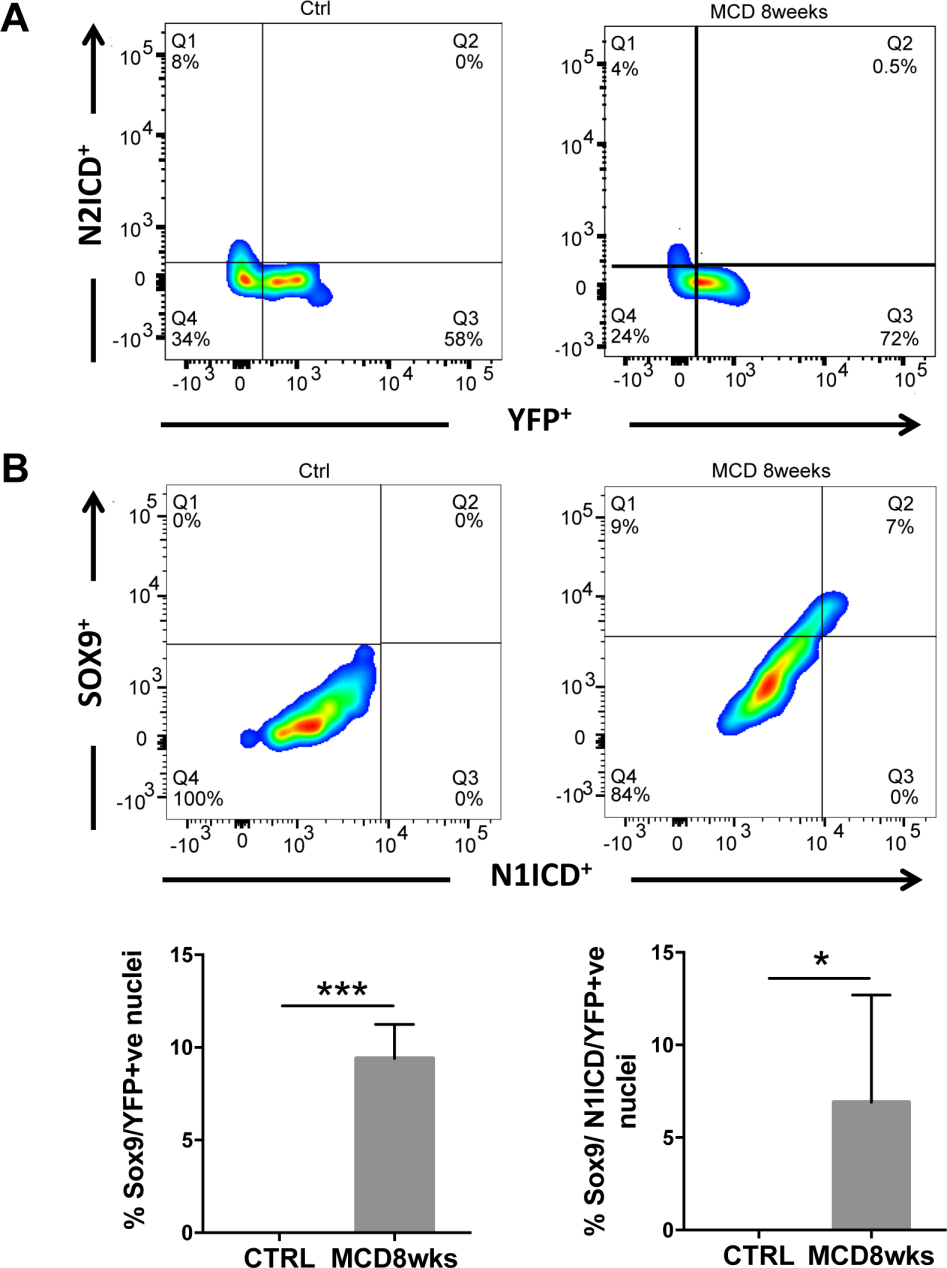
Notch is active in Sox9 positive hepatocytes in MCD diet-injured livers. Nuclei were isolated from paraffin liver sections of R26R-YFP mice infected with AAV8-TBG-Cre (untreated or treated with MCD diet for 8 weeks) to perform FACS analysis. DAPI/YFP+ve nuclei (hepatocytic population) were gated. **(A)** As shown in the representative FACS plot, YFP+ve nuclei were negative for N2ICD staining both in control and MCD treated mice. **(B)** On the contrary, a sub-population of YFP+ve nuclei were positive for Sox9 and the majority of them were also co-expressing N1ICD. The bar graphs show the quantification of FACS analysis results. (*p<0.05; ***p<0.001).

First, we analysed by immunohistochemistry the expression of the Notch-regulated gene Sox9, normally expressed by HPC and mature biliary cells. We found that after 8 weeks of MCD diet, approximately 10% of YFP+ve cells of hepatocellular origin were also Sox9 positive (Fig 6B). This result suggested that Notch signaling might be active in a subpopulation of hepatocytes. Therefore we analysed in hepatocytes YFP/Sox-9+ve the expression of NICD1 and NICD2, the intracellular domains of respectively Notch1 and 2 receptors that normally translocate to the nucleus and bind RBPjk following Notch signaling activation. Intact nuclei were isolated from paraffin embedded livers of R26R-YFP reporter mice (untreated controls and treated with MCD for 8 weeks) and were selected by flow cytometry, based on the co-expression of YFP-Sox9 with NICD1 and NICD2. We found that while NICD2 was not expressed in YFP+ve nuclei (Fig 7A), more than 6% of them were co-expressing NICD1 and Sox9 (Fig 7B). Also by FACS analysis we further confirmed that 10% of YFP+ve nuclei were expressing Sox9 (Fig 7B).

Thus, we hypothesized that HPC infiltrating the lobule and adjacent to fat-laden hepatocytes may actually derive from a Notch-mediated reprogramming of hepatocytes exposed to the pathologic environment of steatohepatitis. To confirm that hepatocytes can transdifferentiate into HPC during MCD diet-induced liver damage, we analysed by immunohistochemistry the distribution of CK19 in R26R-YFP reporter mice treated with MCD diet for 8 weeks. We found that more than 2% of CK19+ve cells were also YFP+ve (Fig 6C), consistent with the hypothesis that a subpopulation of Sox9+ve hepatocytes may eventually transdifferentiate into HPC/DR as also indicated by the HPC-like morphology of CK19+ve/YFP+ve cells. Of note, CK19+ve/YFP+ve and Sox9+ve/YFP+ve cells were strictly localized in lobular areas (*not shown*). These results strongly indicate that CK19+ve cells infiltrating the liver lobule in steatohepatitis partially derive from hepatocytes reprogramming, occurring after Notch-1 induced Sox9 expression.

#### Notch induces phenotypic changes in the hepatocytic cell line AML-12

**Fig 8:**
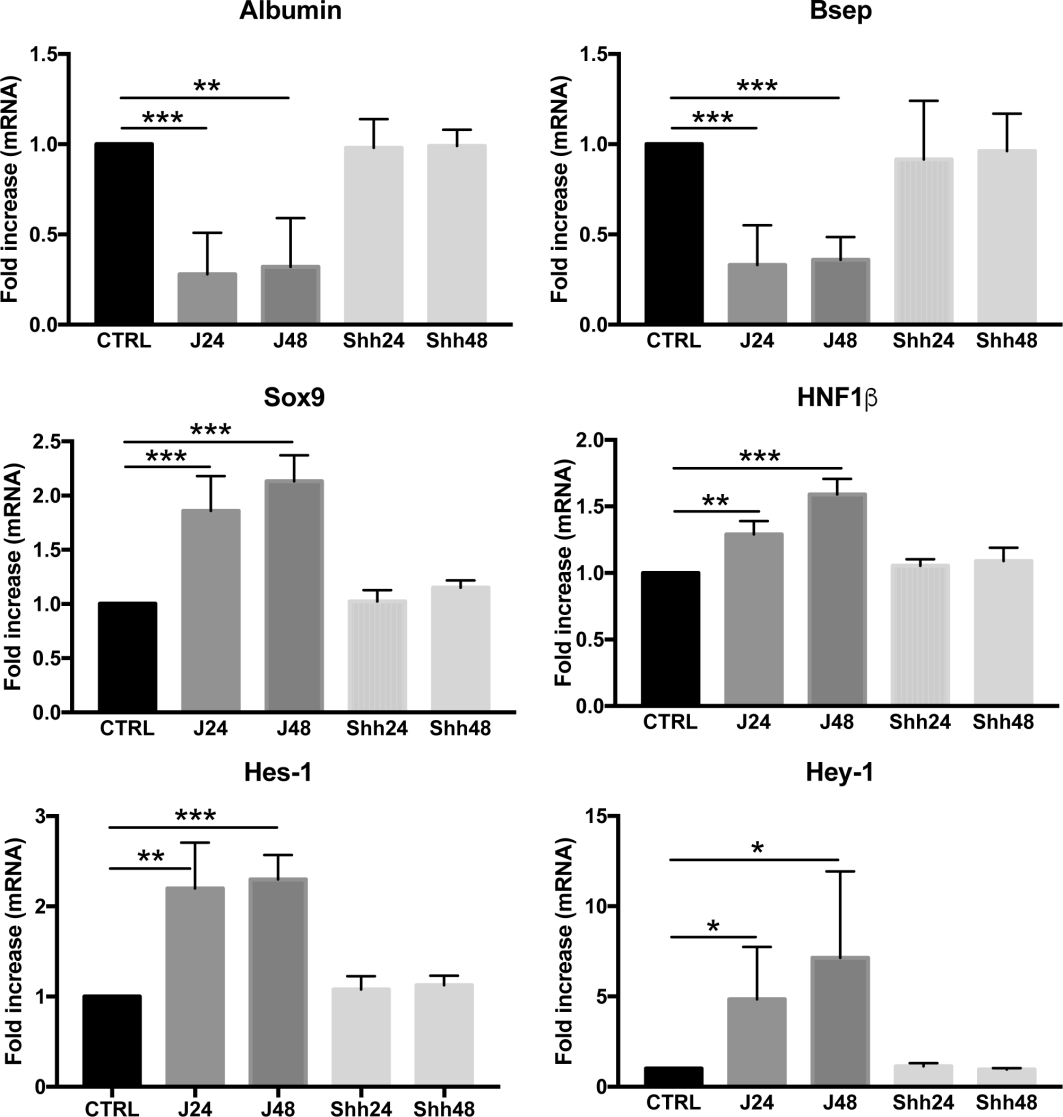
Notch induces phenotypic changes in the hepatocytic cell line AML-12. AML-12 cells were treated with either Jag1 or Shh for 24hours and 48hours to induce respectively Notch and Hedgehog activation. The activation of Notch signaling reduced the expression of markers of hepatocytes (albumin and Bsep), while rapidly increasing the expression of Sox9. The increased expression of Notch target genes Hes1, Hey1 and Hey2 in Jag1 treated cells confirmed that this was a Notch-dependent process. No significant changes in gene expression were detected in the cells treated with Shh at any time point. Data are expressed as fold increase/decrease respect to control (n = 3–4 experiments; *p<0.05, **p<0.01, ***p<0.001).

To further establish the role of Notch in cellular reprogramming toward the HPC-like phenotype, we tested the effects of Notch activation in hepatocytes *in vitro*. To mimic Notch activation we cultured AML-12 cells onto immobilized Jag1, to reproduce the nature of cell-cell contact. As shown in Fig 8, continuous Notch stimulus in AML-12 cells strongly reduced the expression of albumin and the expression of bsep/abcb11, a bile acid transporter unique to hepatocytes, consistent with the overall down-regulation of hepatocytic markers.

Conversely, the biliary markers Sox9 and HNF1β and Notch target genes Hes-1 and Hey-1 were significantly induced after Jag1 treatment in AML-12 cells (Fig 8), supporting a phenotypic change toward the biliary lineage in response to Notch activation.

Sox9 may also be a downstream effector of Hedgehog signaling[29]. Therefore we compared the gene expression of the above markers in AML-12 cells that were treated with recombinant Sonic hedgehog ligand (Shh). As shown in Fig 8, direct activation of Hedgehog signaling did not change the gene expression of either Sox9 or other biliary and hepatocytic markers, suggesting that Hedgehog signaling is unlikely involved in this process.

#### Activated hepatic stellate cells (HSC) express high levels of Jag1, which may contribute to Notch-activation in hepatocytes

**Fig 9:**
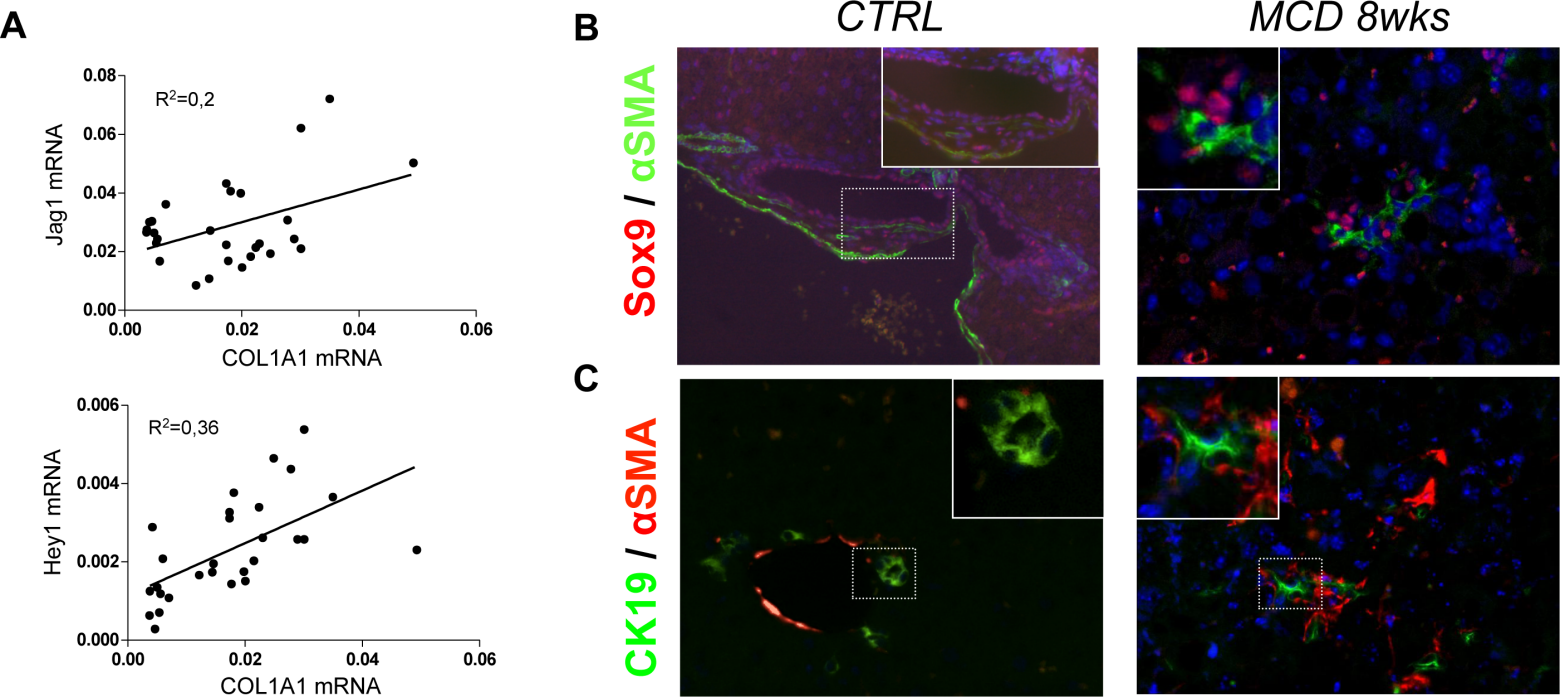
The fibrotic environment is linked to Notch activation in hepatocytes in MCD diet-treated mice. The hepatic expression of Jag1 and Hey1 mRNA positively correlated with that of COL1A1 **(A)**, suggesting a link between the fibrotic environment and Notch pathway. Moreover, in mice treated with MCD diet for 8 weeks, Sox9+ve hepatocyte-like cells (green) established close contact with αSMA+ve cells (red) **(B)**; accordingly, αSMA+ve cells (red) localized in proximity of CK19+ve cells (green) **(C)**, indicating HSC/hepatocytes cross-talk thus suggesting HSC involvement in persistent stimulation of Notch receptors on hepatocytes. Original magnification: 200X, insets 400X.

**Fig 10:**
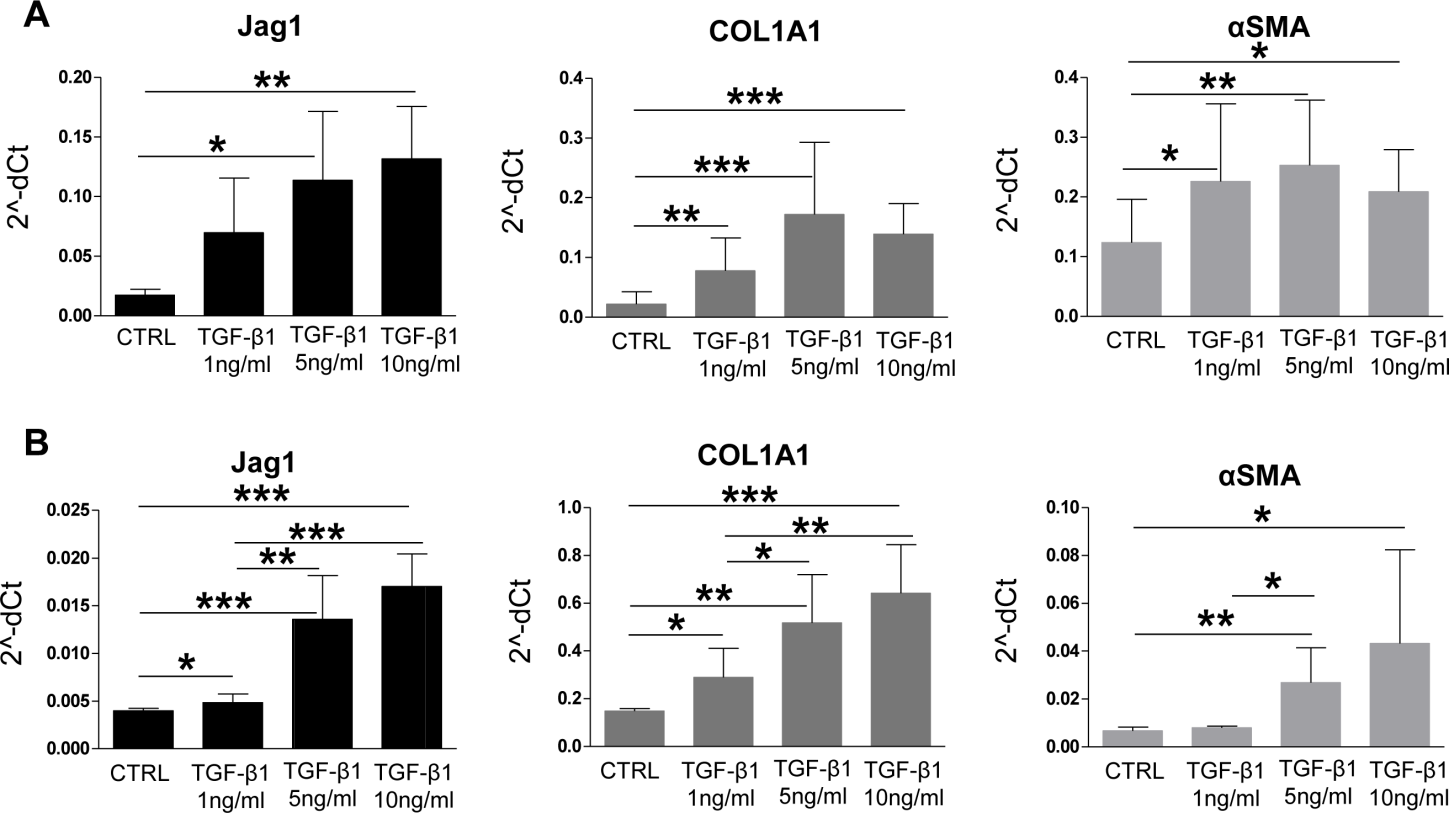
HSC express high levels of Jag1 after TGF-β1-induced activation *in vitro*. Jag1 was strongly upregulated in primary mouse HSC **(A)** as well as in the LX2 cell line **(B)**, when treated with increasing doses of TGF-β1 (1-5-10ng/mL) for respectively 4 days or 72hrs; COL1A1 and αSMA expression confirmed TGF-β1-induced HSC activation **(C)**. (n = 3–15 experiments; *p<0.05, **p<0.01; ***p<0.001).

Notch-induced hepatocytes reprogramming occurring in a MCD diet-treated mouse is most likely a ligand-dependent process, as confirmed by Notch-activation assays *in vitro*. Therefore we sought to investigate which cell type was responsible of providing persistent Jag1-driven stimulus to hepatocytes. Hepatic gene expression of the ligand Jag1 and of the Notch target gene Hey1 positively correlated with COL1A1 expression (Fig 9). Moreover, in MCD diet-fed mice, αSMA immunoreactive cells were localized around Sox9+ve hepatocytes (Fig 9B) as well as in close contact with CK19+ve cells (Fig 9C). All together these results suggest that HSC/myofibroblasts could stimulate continuous Notch activation in hepatocytes. As shown in Fig 10, in response to increasing doses of TGF-β1, HSC up-regulated Jag1 expression in a dose-dependent manner, consistent with the hypothesis that HSC are probably the cells transmitting the Notch signal to hepatocytes during NASH. Consistent with this interpretation, fibrosis increased linearly with the increase in HPC/DR area (CK19+ve), and the HPC/DR area positively correlated with TGF-β1 and COL1A1 (Fig 2).

#### Notch signaling inhibition by DBZ reduces HPC response and the progression of steatohepatitis

**Fig 11:**
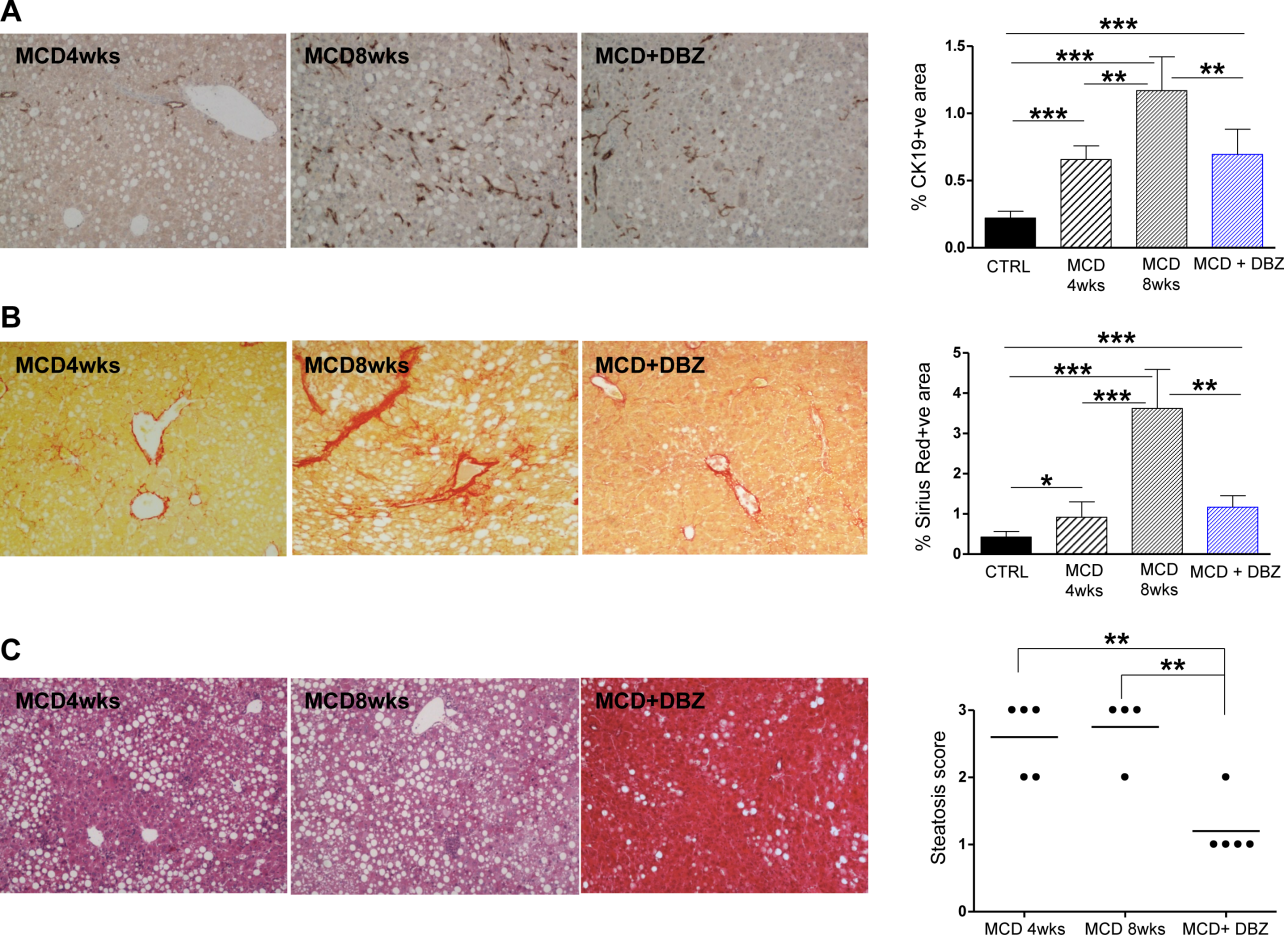
Notch inhibition reduces HPC expansion and ameliorates steatohepatitis in MCD diet-fed mice. Immuno-peroxidase staining for CK19 showed a significant reduction in HPC/DR activation after Notch inhibition, as demonstrated by morphometric quantification reported in the bar graph **(A)**. In DBZ-treated mice with steatohepatitis, also the progression of liver fibrosis was attenuated: morphometric quantification confirmed that the percentage of the area positive for Sirius red was comparable to that reported in the group fed with MCD diet for 4 weeks **(B)**. H&E staining shows reduction of steatosis after Notch inhibition, as confirmed by steatotic score quantification **(C)**. (n = 4–5 mice per group; *p<0.05, **p<0.01; ***p<0.001). Original magnification: 100X.

To evaluate the effects of Notch inhibition during steatohepatitis, after 4 weeks of MCD diet (when steatohepatitis was established in mice) we started daily DBZ administration along with MCD treatment. Notch inhibition in mice with steatohepatitis decreased HPC/DR expansion induced by MCD diet (Fig 11A) and the number of hepatocytes Sox9 positive (S1 Fig), suggesting a role of Notch in this mechanism. DBZ treatment also inhibited fibrosis progression: collagen deposition in these mice was comparable to that reported in mice fed MCD diet for 4 weeks, and significantly lower than fibrosis evolution reported after 8 weeks of MCD diet (Fig 11B). Moreover, inhibition of Notch significantly attenuated steatosis score (Fig 11C). Together these data support the observation that downregulation of Notch receptors is a physiologic response to counterbalance fat accumulation in hepatocytes.

## Discussion

Increasing evidence indicates that HPC activation contributes to disease progression in a number of chronic liver diseases. Steatohepatitis can be associated with alcoholic liver disease, metabolic liver disease and several other genetic an acquired liver disorders[30–32], Understanding HPC/DR pathobiology is fundamental to unravel the mechanisms leading to cirrhosis and liver cancer. We have observed that steatohepatitis induced by MCD diet is associated with diffuse HPC/DR expansion not only in the portal areas but also into the liver lobule, where HPC establish intimate contacts with fat-laden hepatocytes. Prior work has shown that HPC are under the control of a number of developmental mechanisms, including Hg, Wnt and Notch signaling, which is necessary to modulate morphogenesis during repair from biliary damages[4, 11]. We have previously shown the pivotal role of Notch in biliary repair and tubulogenesis[11]. Here we show that in the MCD diet-induced model of steatohepatitis, HPC/DR activation correlates with inflammation and fibrosis (Figs 1 and 2), and that HPC are spread into the liver parenchyma in close contact with fat-laden hepatocytes and activated myofibroblasts.

HPC/DR are believed to arise from the hepatic stem cell niche located in the canals of Hering and from the small ductules of the biliary tree[33, 34], but their origin has not been fully elucidated. In fact, recent reports indicate that under certain conditions, HPC/DR may also be generated from the biliary transdifferentiation of hepatocytes (biliary metaplasia)[21, 35–37]. Our data are consistent with this interpretation, but provide evidence that only a subset of CK19+ve cells present in the lobule is derived from hepatocytes that underwent Notch-dependent phenotypic changes.

Given its relevance in regulating cell fate in general and HPC/DR response in liver disease[10, 11], it is not surprising that Notch signaling promotes HPC/DR expansion in the MCD diet-induced steatohepatitis. Even considering the possible non-specific effects of γ-secretase inhibitors, pan-Notch inhibition dibenzazapine (DBZ) significantly reduced the extent of the HPC/DR response.

Our data suggest that in steatohepatitis Notch activation is cell specific. Using whole liver lysates and tissue from laser capture microscope, we found that gene expression of Notch signaling components remained in CK19+ve cells, but was significantly down-regulated in CK19-ve cells. Prior work from Pajani et al reported activation of Notch signaling in the liver of mice with obesity and fatty liver[24, 25]; however, in this model steatosis was associated to insulin resistance, but lacked appreciable inflammation/fibrosis. On the contrary, the MCD model (as shown in Fig 2) is characterized by steatohepatitis and fibrosis, without obesity and insulin resistance[20]. In this model, the down-regulation of hepatocellular Notch receptors/ligands during chronic liver damage may have a protective role. In fact, in mice treated with MCD diet and DBZ, steatosis, fibrosis and HPC/DR were reduced (see Fig 11).

In our conditions, in spite of downregulation of Notch receptors and ligands in hepatocytes (CK19-ve fraction), the Notch downstream signal was maintained. In fact, in the CK19–ve areas, the lower expression of Numb (an endogenous inhibitor of Notch) would, actually make hepatocytes more prone to Notch activation. Furthermore, *in vivo* hepatocyte lineage tracing experiments showed that after 8 weeks of MCD diet a subset of hepatocytes (YFP+ve) expresses Sox9, a Notch target gene involved in biliary differentiation and liver carcinogenesis[9, 16]. Although Sox9 expression is also regulated by Hg[29], these data suggest that in a subpopulation of hepatocytes Notch signaling is actually activated. Consistent with this interpretation, we found that most of the YFP+ve/Sox9+ve were also positive for NICD1, the active domain of Notch1. Finally, we also detected CK19 expression in YFP+ve cells with a morphology resembling smaller HPC-like cells. These results suggest that in mice with steatohepatitis, a slow step-wise process induces hepatocytes conversion to HPC-like cells, contributing to DR as disease progresses. Of note, we have previously shown that in AGS, there is an accumulation of hepato-biliary intermediate cells that are not able to completely commit into functional cholangiocytes[12], supporting the idea that hepatocytes might undergo phenotypic conversion to sustain HPC-driven repair, and this requires Notch signaling for a full differentiation[21]. Sox9 expression in hepatocytes could therefore represent the starting point of Notch-mediated reprogramming occurring in disease conditions.

*In vitro* studies with an hepatocytes cell line provided further evidence that Notch activation in hepatocytes leads to cellular reprogramming driving the expression of biliary markers along with decreasing hepatocytic features. Interestingly, Sox9 was rapidly induced by Jag1-dependent Notch activation but not by the Hedgehog ligand Shh. Altogether these results highlight the relevance of Sox9 appearance in hepatocytes in order to initiate phenotype conversion. Sox9 expression has been reported in a subset of HCC and linked to a more malignant and aggressive phenotype[16]. Since HCC is a known complication of NASH, we speculated that Sox9 expression could represent an early step in NASH-related hepatocarcinogenesis. Of note, Villanueva and colleagues[16] reported that experimental persistent Notch activation in the hepatic compartment led to HCC after 12 months with 100% penetrance, suggesting that aberrant and continuous Notch activation in hepatocytes drives HCC development.

We next sought to determine which cell type could provide continuous Notch activation in hepatocytes in steatohepatitis, given that in hepatocytes Jag1 expression is actually downregulated. We reasoned that the likely source of Notch ligands could be myofibroblasts, that are known as the main contributors to liver fibrosis in MCD diet[38]. Given their intimate contact with hepatocytes and HPC, myofibroblasts could also explain why Notch would be activated only in a subset of hepatocytes. Moreover, our data showed that hepatic expression of Jag1 and Hey1 correlated with that of collagen. Consistent with this hypothesis, we showed that treatment with the pro-fibrogenic factor TGF-β1 strongly induced a dose-dependent expression of Jag1 in primary isolated HSC and in a human HSC cell line, indicating HSC as a likely source of Jag1 in fibrotic settings. We also showed that indeed in MCD diet-fed mice, activated-HSC spread throughout liver parenchyma in close contact with both fat-laden hepatocytes and CK19+ve cells (Fig 9C). Activated-HSC localized around Sox9+ve hepatocyte-like cells (Fig 9B), supporting the idea that HSC could potentially be responsible of persistent Notch stimulus in hepatocytes. Involvement of Notch signaling in these mechanisms is further demonstrated by the significant reduction in both HPC/DR and fibrosis in MCD diet-fed mice treated with DBZ (Fig 11A and 11B).

Our results indicate that Notch signaling is involved in the generation of HPC/DR response in steatohepatitis, by mediating a cross-talk between liver cells and mesenchymal cells able to mobilize the HPC niche and also to facilitate hepatocyte trandifferentiation into HPC/DR. In fact, myofibroblasts stimulated by TGF-β1, upregulate Jag1 that in turn stimulates Notch-dependent Sox9 expression in adjacent hepatocytes, eventually leading to hepatocytes reprogramming into HPC/DR. Further studies will clarify the role of these mechanisms on liver carcinogenesis in steatohepatitis, as well as the possible therapeutic implications of these findings.

## Supporting information

S1 Fig Notch inhibition reduces Sox9 expressing hepatocytes in MCD diet fed mice. Bar graph shows a significant reduction in the percentage of Sox9+ve hepatocytes in MCD diet fed mice treated with DBZ. (n = 4–5 mice per group; ***p<0.001).
(TIF)
