# Supplementary figures and images for: Notch signaling and progenitor/ductular reaction in steatohepatitis
Source: PLoS One. 2017 Nov 15;12(11):e0187384. doi: 10.1371/journal.pone.0187384 (PMC5687773; doi:10.1371/journal.pone.0187384)

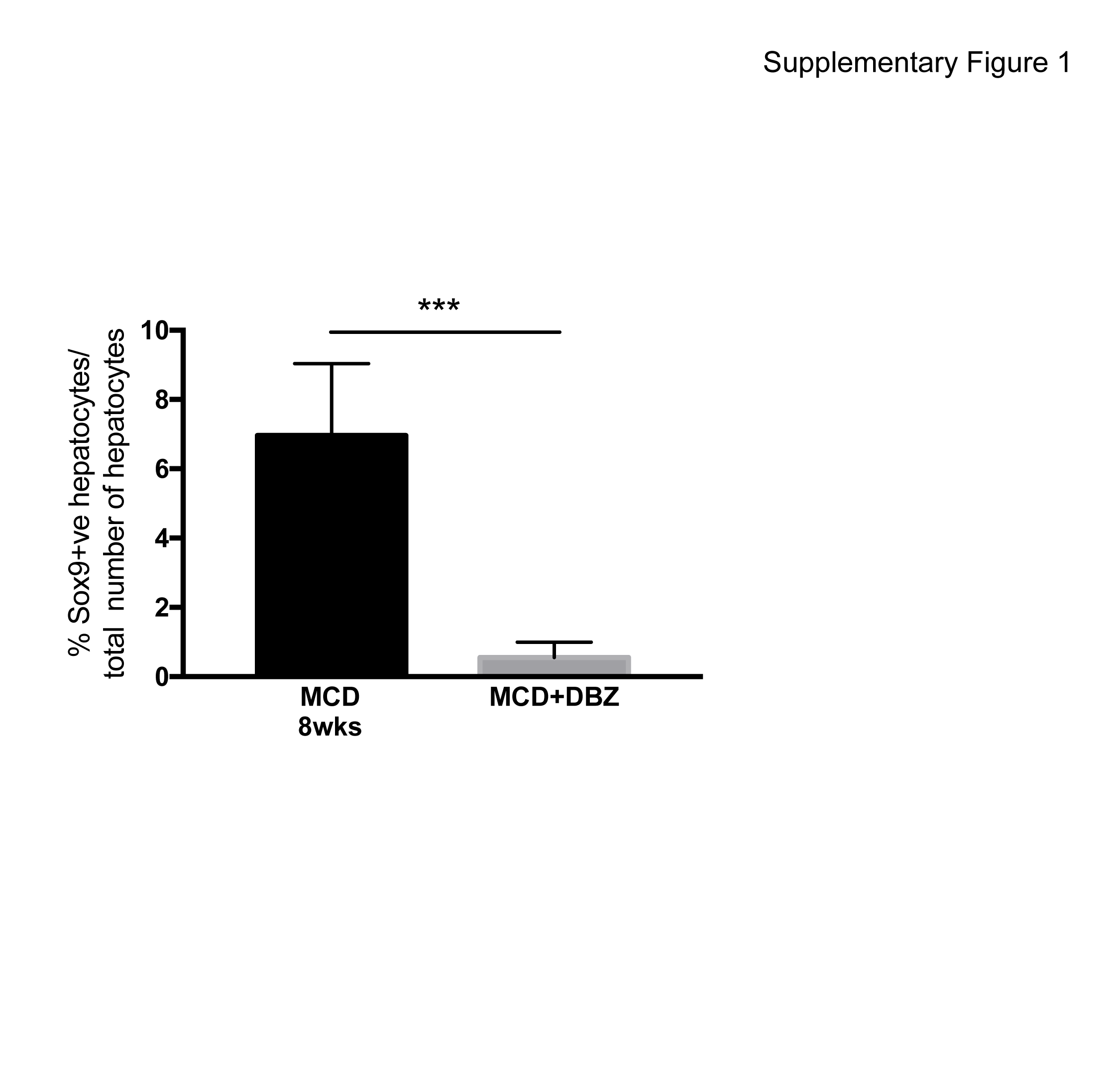

Supplement: S1 Fig — Bar graph shows a significant reduction in the percentage of Sox9+ve hepatocytes in MCD diet fed mice treated with DBZ. (n = 4–5 mice per group; ***p<0.001). (TIF) [file pone.0187384.s001.tif]
